# Supplementary material for: Content-rich biological network constructed by mining PubMed abstracts
Source: BMC Bioinformatics. 2004 Oct 8;5:147. doi: 10.1186/1471-2105-5-147 (PMC528731; doi:10.1186/1471-2105-5-147)
Supplement: Additional File 2 — The original results of the above study (non-essential files are deleted to keep the file size under the limit set by BMC bioinformatics). [file 1471-2105-5-147-S2.bz2 › chilibotAdditionalFile2/dip05/622ID10417423E11008/html/CAPN2_CAPNS1.html]

 


 **CAPN2** and **CAPNS1** 
  
Found 230 abstracts in PubMed, retrieved 05.  
 

 What does Google say? 
 PDF only 
| .edu only 

---

**Interactive relationship** (e.g. stimulation, inhibition, etc)

**Non-interactive relationship** (e.g. studied together, co-existance, homology, etc.)

- In VANP and CANP  [ **CAPNS1** ]   [ **CAPN2** ] , a higher cytokine content was associated with neuropathic pain.  Ref: 12734666 Acta Neuropathol Berl , 2003
- We used immunohistochemistry including double labeling and morphometry to localize and quantify the expression of interleukin 1 beta IL 1beta, IL 6, and tumor necrosis factor alpha TNF in sural nerve biopsy samples of 41 patients with vasculitic neuropathy VANP, chronic inflammatory demyelinating neuropathy CIDP, non inflammatory chronic axonal neuropathy CANP  [ **CAPNS1** ]   [ **CAPN2** ] , and 3 controls.  Ref: 12734666 Acta Neuropathol Berl , 2003
- The selective NPR C receptor agonist, cANP  [ **CAPNS1** ]   [ **CAPN2** ]  4 23, mimicked ANF response in a dose dependent fashion.  Ref: 12829435 Am J Physiol Gastrointest Liver Physiol< MedlineTA>Am J Physiol Gastrointest Liver Physiol, 2003
- The enzyme is a heterodimer, encoded by the genes  **capn2** , for the large subunit, and capn4  [ **CAPNS1** ] , for the small subunit.  Ref: 12559913 J Mol Biol, 2003
- Overall cytokine immunoreactivity was highest in VANP, less strong in CIDP and lowest in CANP  [ **CAPNS1** ]   [ **CAPN2** ] .  Ref: 12734666 Acta Neuropathol Berl , 2003
- By employing this method, CANP  [ **CAPNS1** ]   [ **CAPN2** ]  activity in different regions of rat brain was determined.  Ref: 12385765 J Neurosci Methods, 2002
- The utility of the method was evaluated by assaying the proteolytic activities of trypsin and calcium activated neutral protease CANP  [ **CAPNS1** ]   [ **CAPN2** ] .  Ref: 12385765 J Neurosci Methods, 2002
- The rate of gastric emptying was related to CANP  [ **CAPNS1** ]   [ **CAPN2** ]  but not gastric symptoms or actual glycemic control.  Ref: 12600185 Pol Arch Med Wewn, 2002
